# EP-DNN: A Deep Neural Network-Based Global Enhancer Prediction Algorithm

**DOI:** 10.1038/srep38433

**Published:** 2016-12-08

**Authors:** Seong Gon Kim, Mrudul Harwani, Ananth Grama, Somali Chaterji

**Affiliations:** 1 Department of Computer Science, Purdue University West Lafayette, Indiana, USA.

## Abstract

We present *EP-DNN*, a protocol for predicting enhancers based on chromatin features, in different cell types. Specifically, we use a deep neural network (DNN)-based architecture to extract enhancer signatures in a representative human embryonic stem cell type (H1) and a differentiated lung cell type (IMR90). We train EP-DNN using p300 binding sites, as enhancers, and TSS and random non-DHS sites, as non-enhancers. We perform same-cell and cross-cell predictions to quantify the validation rate and compare against two state-of-the-art methods, DEEP-ENCODE and RFECS. We find that EP-DNN has superior accuracy with a validation rate of 91.6%, relative to 85.3% for DEEP-ENCODE and 85.5% for RFECS, for a given number of enhancer predictions and also scales better for a larger number of enhancer predictions. Moreover, our H1 → IMR90 predictions turn out to be more accurate than IMR90 → IMR90, potentially because H1 exhibits a richer signature set and our EP-DNN model is expressive enough to extract these subtleties. Our work shows how to leverage the full expressivity of deep learning models, using multiple hidden layers, while avoiding overfitting on the training data. We also lay the foundation for exploration of cross-cell enhancer predictions, potentially reducing the need for expensive experimentation.

Cell types are unique, in spite of the fact that they contain the same genomic DNA, largely because of their differential gene expression patterns. This in turn is a function of the regulatory genomic regions—specialized cis-regulatory modules (CRMs), such as, enhancers^1^, silencers, promoters, and insulators^2^,^3^,^4^. Among these, genomic enhancers constitute a prominent class of CRMs, often located far from the gene promoters that are responsible for mediating gene transcription^5^. Enhancers can be defined as short DNA sequences regulating temporal and cell-type specific basal gene-transcription levels, from transcription start sites (TSSs), at distances ranging from hundreds of bases to, in rare cases, even megabases^6^,^7^,^8^. Knowing their properties, regulatory activity, and genomic targets is crucial to the functional understanding of cellular events, ranging from cellular homeostasis to differentiation. Recent genome-wide investigation of epigenomic marks has indicated that enhancer elements could be enriched for certain epigenomic marks, such as complex, albeit predictive, combinatorial histone modifications. Our efforts in this paper are motivated by these recent advances in epigenomic profiling methods, which have uncovered enhancer-associated chromatin features in different cell types and organisms^9^,^10^,^11^,^12^. Specifically, in this paper, we use recent state-of-the-art deep learning methods and develop a deep neural network (DNN)-based architecture^13^,^14^,^15^ to predict the presence and types of enhancers in the human genome, “learning” from the combinatorial histone modification codes. We call our system “**EP-DNN**”, an acronym for “**E**nhancer **P**rediction using **D**eep **N**eural **N**etwork”.

Computational identification of enhancers has proven challenging due to several reasons^16^. First, the search space of genomic enhancers is large. Second, while enhancers regulate genes in *cis*, they do not display distinct locational or orientation-centric signals relative to the genes that they regulate^17^. This is because enhancers can function at a distance from their target genes via chromatin looping that bring the enhancers and target genes in three-dimensional proximity^18^,^19^. Alternately, enhancers can function via direct eRNA transcription from the enhancer DNA sequences^20^.

Several high-throughput experimental approaches exist to identify enhancers^21^,^22^. The first is mapping specific transcription factor (TF) binding sites (TFBS) through ChIP-seq^22^. This stems from the fact that short enhancer DNA sequences serve as binding sites for TFs, and the combined regulatory cues of all bound TFs determine ultimate enhancer activity^23^,^24^. However, this approach requires the knowledge of the TF combinations that are expressed and occupy binding sites in a specific physiologic setting^25^. Therefore, predicting enhancer activity from sequence-based information, such as from the TF motif content, remains challenging^24^,^26^. The second is based on mapping transcriptional co-activator binding sites (e.g., histone acetyltransferase HAT, *p300*)^27^,^28^. However, not all enhancers are marked by a set of co-activators. The third approach relies on identifying DNase-I hypersensitivity (DHS) sites^8^. However, DHS sites lack specificity because DNase-I can map to other CRMs as well, as evident from our ground truth diagram. Finally, the fourth approach involves histone modification patterns produced by ChIP-seq that consistently mark enhancer regions^29^,^30^,^31^,^32^,^33^, and which thus is our method of choice in this paper.

## Related Work

Several computational methods that use histone modification signatures to identify enhancer regions have been developed. Won *et al*. proposed the use of Hidden Markov Models (HMMs) to predict enhancers using three primary histone modifications^30^. Firpi *et al*. focused on the importance of recognizing the histone modification signals through data transformation and employed Time-Delayed Neural Networks (TDNNs) using a set of histone marks selected through simulated annealing^31^. Fernández *et al*. used Support Vector Machines (SVMs) on an optimized set of histone modifications found through Genetic Algorithms^32^. RFECS (Random Forest based Enhancer identification from Chromatin States) improved upon the limited number of training samples in previous approaches using Random Forests (RFs), in order to determine the optimal set of histone modifications to predict enhancers^33^. We provide a comparison of some of the recent methods of enhancer prediction in Table 1, comparing the following enhancer prediction protocols: RFECS^34^, DEEP-ENCODE^35^, ChromaGenSVM^32^, CSI-ANN^31^, and HMM^30^.

In addition to histone modifications, recent work has also used other input features to classify regulatory sites in DNA. For example^36^, is a complementary line of work in which the authors further classify enhancers as strong or weak enhancers. For their input features, they use k-mers of DNA nucleotides, while we use histone modification patterns. The results are not directly comparable to ours because their ultimate classification task is also different. Further, looking at a finer level of detail, their classification ignores whether an enhancer is poised or active, and considers the simpler, two-way classification of strong or weak enhancers. Another recent paper shows how to input biological sequences into machine learning algorithms^37^. The difficulty arises from the fact that ML algorithms need vectors as inputs and a straightforward conversion of the biological sequence into a vector will lose important information, such as ordering effect of the basic elements^38^ (C for DNA, amino acids for protein). Prior work developed the idea of generating pseudo components from the sequences that can be fed into the ML algorithm. The above-mentioned paper unifies the different approaches for generating pseudo components from DNA sequences, RNA sequences, and protein sequences. This is a powerful and general-purpose method. In our work, however, we do not need this generality. We feed the (24 different) histone modification markers and, by binning, we consider features corresponding to adjacent genomic regions for each marker (20 for each histone modification marker). We shift the window gradually thus capturing the overlapping regions among contiguous windows and the DNN extracts the relevant ordering information, thanks to such overlap. Further in repDNA^39^, the authors consider DNA sequences alone. RepDNA calculates a total of 15 features that can be fed into ML algorithms. The 15 features fall into 3 categories—nucleic acid composition, autocorrelation features describing the level of correlation between two oligonucleotides along a DNA sequence in terms of their specific physicochemical properties, and pseudo nucleotide composition features.

## EP-DNN’s Contributions

In this paper, we solve the classification problem of whether a histone combinatorial code represents an enhancer element, or not, using our deep learning-based classifier, EP-DNN. Our main contributions in this paper are as follows:

- We have developed an efficient DNN-based classifier to identify enhancers in two *distinct* cell types, namely the human embryonic stem cell type (H1) and a differentiated primary lung fibroblast cell line (IMR90). We demonstrate that DNNs work well in extracting features in a high-dimensional space (a set of eighty features coming from four histone modifications), and then, in predicting enhancers. A DNN has a large configuration space and we take care to avoid overfitting on the training data. Our technique is able to identify multiple kinds of enhancers, even when trained on only a single kind (p300).
- When keeping the number of enhancer predictions by RFECS, DEEP-ENCODE (DEEP-EN), and EP-DNN equal for purposes of comparison (roughly at 100,000 predictions), we find that our protocol has superior accuracy, specifically, with a validation rate of 91.6%, for same-cell and cross-cell predictions, relative to 85.3% for DEEP-EN and 85.5% for RFECS. EP-DNN appears to provide a more powerful model, potentially capturing the more tightly-packed, albeit richer, data embedded in the embryonic H1 cell type. This is because while the H1 dataset appeared to be a more complex dataset to train on, it also achieved higher accuracy on same-cell (H1 → H1) or cross-cell (H1 → IMR90) predictions. Thus, EP-DNN demonstrates further improvement in IMR90 prediction results when H1 is used as the training set, with validation rates of 95.40% (EP-DNN) compared to 93.00% (RFECS) and 81.42% (DEEP-EN). This finding also hints at the global enhancer prediction capabilities of our EP-DNN, i.e., prediction across cell types, potentially reducing the need for experimental results on new cell types.
- EP-DNN has lower computational cost when compared to some of the state-of-the-art enhancer prediction methods. At the upper end of the prediction set size, with 40,000 samples: RFECS took 30 seconds, DEEP-EN took 15 seconds, while EP-DNN took less than 2 seconds. The slope of the running time is also the lowest for EP-DNN, which indicates that our method will scale well for larger numbers of predictions.

## Materials and Methods

**Figure 1:**
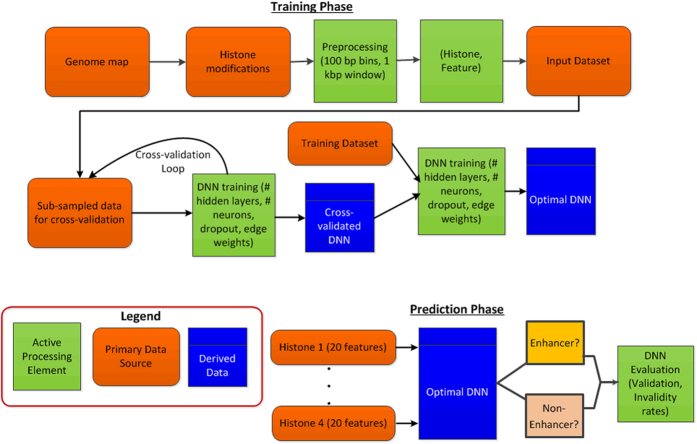
Overview of our solution approach in which we train DNNs using the histone modifications and their associated features. We perform weight analysis and feature selection to identify the optimal DNN, which is then used for predicting if a regulatory region is an enhancer or not.

We present a high level overview of our approach in Fig. 1. In the figure, we show, separately, the training phase and the prediction phase. In the training phase, we create an optimal DNN using a set of histone modifications and the associated spatial features and, in the prediction phase, we use the same set of features to predict if a regulatory region is an enhancer or not, followed by validation of our results. We predict enhancers in two distinct *human* cell types—embryonic stem cells (H1) and primary lung fibroblasts (IMR90), which were generated as a part of the NIH Epigenome Roadmap Project^12^.

**Figure 2:**
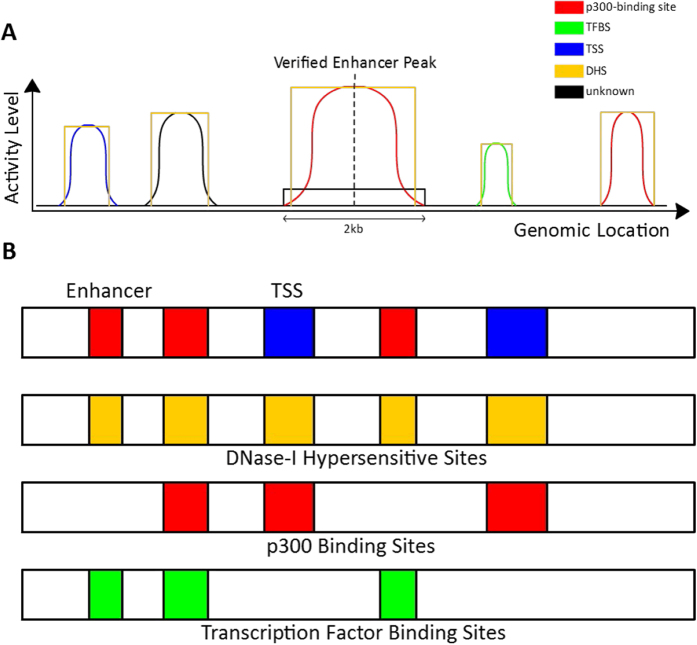
A notional schematic showing the enhancer and the TSS (the promoter) relative to some of the True Positive Markers (TPMs) ─ DNase-I hypersensitivity site (DHS), p300 binding site, and transcription factor binding site (TFBS) (applicable to the H1 cell line). Various forms of these TPMs overlap with the enhancer and the promoter sites. An overlap of the DHS with the TFBS can indicate an enhancer, while an enhancer is typically distal to the TSS. TPMs refer to DHS, p300, CBP, and TFBS.

To train our DNN, we first select distal p300 co-activator binding sites through ChIP-seq, and then further select the regions representing enhancers through overlapping these p300 sites with DHS that are distal to TSS. These serve as our positive training examples. For negative training examples, representing non-enhancers, we select TSS that overlap with DHS, as well as random 100 bp bins that are distal to known p300 or TSS. The corresponding histone modification signatures of our collected sites are then used as input to a DNN. Figure 2 gives a schematic, indicating the rationale used to map enhancers and TSS sites in relation to the different true positive markers (TPMs) used in our method.

### Datasets

The development of EP-DNN is motivated by the availability of data from large scale projects, such as the ENCODE project^9^, which has annotated 400,000 putative human enhancers, with the current estimate of enhancer numbers being over a million^40^. Another extensive database is the NIH Roadmap Epigenomics Project^10^,^12^ that also provides publicly-available epigenomics maps, complementary to ENCODE. In addition, the NCBI’s Gene Expression Omnibus (GEO) repository^11^ also hosts much previous work and data on enhancer prediction. We have used data from all three of these repositories for arriving at our training and validation data for EP-DNN.

p300, and related acetyltransferases, are transcriptional co-activators that bind to TF activation domains and have been found to localize to many active enhancers, but not all^29^. Further, p300 co-activators are ubiquitous, present in all cell types, and control the expression of numerous genes. Therefore, by using p300 enhancer signatures for training, we can also find other types of enhancers (e.g., CBP- or TF-based), generalizing well toward prediction of multiple classes of enhancers. The number of peak call data used is shown in Table 2. Of these, 5,899 p300 peak calls were selected for H1 and 6,000 peak calls from the IMR90 cell line to represent enhancers for the training set. This may appear to be a small fraction of the peaks to use as a training set, but we use this as it reflects the choice of RFECS and thus, our numbers will be comparable.

However, p300 co-activators also bind to Transcription Start Sites (TSS) that are not enhancers. Therefore we include 9,299 TSS peaks from H1 and 8,000 peaks from IMR90 in our training set as negative examples. Finally, 31,994 random distal background sites were selected for H1, and 34,000 for IMR90, to represent non-enhancers, and these also contribute to the negative examples. Logically, Note the p300 sites that were chosen as the positive samples were distal from TSS.

### Histone Modification Inputs, Normalization, Preprocessing

Previous studies indicate H3K4me1, H3K4me2, H3K4me3, and H3K27ac as the top histone modifications^33^ indicative as markers of active enhancers and therefore we selected them for our EP-DNN model. A notional schematic of the enhancer and the TSS (promoter) relative to the various relevant sites—DHS, TFBS, and p300 is given in Fig. 2. The bounding box is the DHS and we are only considering sites that are overlapping with the DHS. The peak location is shown for each element and the activity level curve is shown on both sides of the peak region. The ChIP-seq reads of these histone modifications were binned into 100 bp intervals and normalized against its corresponding inputs by using an RPKM (reads per kilobase per million) measure. We consider a total of 24 histone modification markers corresponding to all the modifications for which data is available in ENCODE and NIH Epigenomics Roadmap Project. For each histone modification, we have 20 features corresponding to 20 windows centered around the peak of the modification activity level. Multiple replicates of histone modifications were used to minimize batch-related differences, and the RPKM-levels of the replicates were averaged to produce a single RPKM measurement per histone modification. The RPKM-levels were further normalized to create a Z-score, based on the mean and the standard deviation of the training set. The transform applied is the standard one *Z* = (*X* − *μ*)/*σ*. The same mean and standard deviation from the training set were also used to normalize the test set before prediction.

### Deep Neural Network (DNN) Model

**Figure 3:**
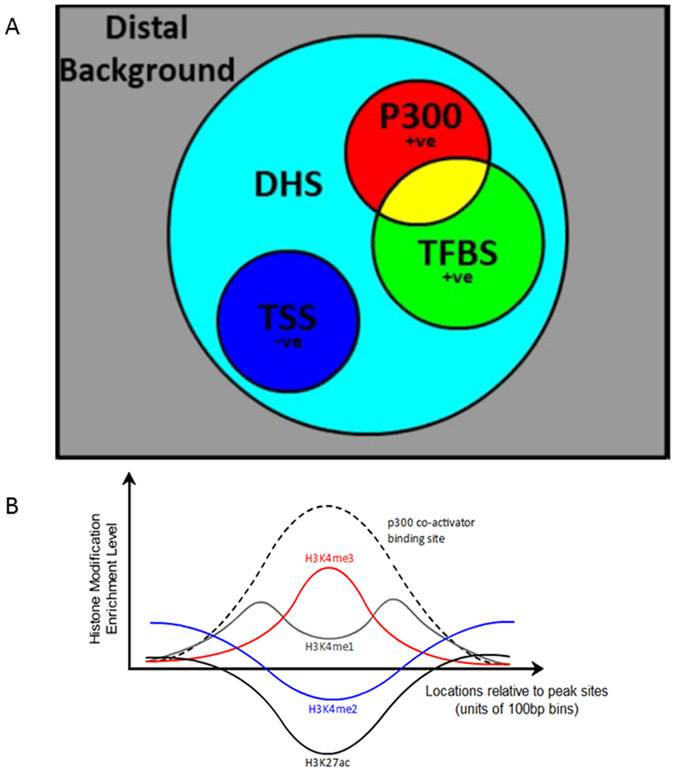
(**A**) The ground truth diagram for the positive and negative examples that EP-DNN uses for H1, with the only caveat being that we use data for Sox2, Oct4, and Nanog, among different possible TFs. For IMR90, again, the ground truth diagram will be similar, just without including the embryonic cell-specific TFs. (**B**) The enrichment level of histone modifications H3K4me1/2/3 and H3K27ac around a p300 co-activator binding site. These histone modification levels are used as input features.

**Figure 4:**
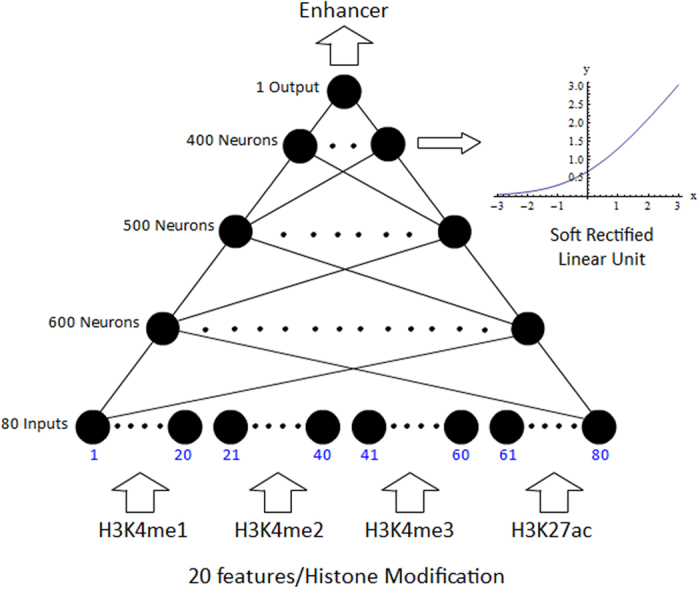
EP-DNN is a fully connected DNN with an 80-600-500-400-1 architecture and *softplus* activation functions. It takes 4 histone modifications (20 features in each mod, with ten 100 bp bins on each side of a location) as input and has a single real valued output which is put through a threshold to determine the classification of a potential enhancer location.

DNNs have the traditional advantage that they provide feature extraction capabilities and do not require manual feature engineering or transformation of the data, which in turn would have required domain knowledge. A fully connected DNN with 80 inputs, 1 output, and *softplus* activation functions for each neuron was used to make enhancer predictions using positive and negative examples, as shown in the ground truth diagram (Fig. 3A), using histone modification combinations as in Fig. 3B. The full architecture of the EP-DNN is shown in Fig. 4. Each input sample consists of four 20-dimensional vectors of 100 bp bin RPKM-levels, windowed from −1 to +1 kb at each bin location. The window is centered at the peak of the different elements (enhancers and non-enhancers). Thus, there is one vector for each of the four histone modifications that we consider, giving a total of 80 input features. Training was done in mini batches of 100 samples via stochastic gradient descent. To prevent overfitting, dropout training^41^ was applied, with a dropout rate of 0.5, along with a weight decay of 0.9. An optimal architecture of three hidden layers, comprising of 600 neurons in the first layer, 500 in the second, and 400 in the third, was found through cross-validation on half the training data, selected randomly. In terms of the hyperparameters, which include the number of layers and number of neurons in each layer, they were tweaked manually through trial and error, for small cross-validation sets. The point was to find a global architecture that matches all cell types extending even to ones not yet analyzed or found, and not overfitting to a specific one.

The full training set was used to train the model and a convergence on the mean squared error was observed with only 5 epochs of training. This extensive training mechanism was found to be suitable to optimize the DNN with its fairly large parameter space.

### Training and Prediction

The DNN was trained with two class values, the selected p300 sites, assigned a value of 1, to represent enhancers, and the TSS and random background sites, assigned a value 0, to represent non-enhances. Two DNN models were built using the same architecture and training method; one trained by data from H1 and the other from IMR90. Note that only the p300 sites, and not the other enhancer types, were used for training as the positive samples. This is because p300 sites are found across different cell types and have been found to generalize well.

Both DNN models were used to make enhancer predictions in H1 and IMR90. Thus, we have four experimental setups.

*Within cell type prediction*: H1 → H1; IMR90 → IMR90

*Across cell type prediction*: H1 → IMR90; IMR90 → H1

Each 100 bp bin in the genome gets a value, which is the output of the DNN. Various threshold values were then applied to the output values to assign each location to an enhancer class, if the value is larger than the applied threshold. If not, the location was assigned to a non-enhancer class. By varying the value of the threshold, we get different values for false positives and false negatives. For comparison against previous algorithms, the same training and testing datasets were applied to RFECS and DEEP-EN for both H1 and IMR90 prediction.

### Measurement of Validation and Invalidity Rates

The standard precision and recall metrics misrepresent actual prediction performance on real data, since there are many more unknown functional sites than just the p300, CBP, NANOG, SOX2, OCT4 binding enhancers, or TSS. Ideally, we would have to evaluate performance on all these sites that are unaccounted for. However, most are not experimentally verified and are unknown. Thus, there is not enough data to make an accurate evaluation of the prediction of any computational model.

Further, functional enhancers are experimentally verified by single peak locations. However, in reality, enhancers exist in various levels (height) and sizes (width) that more or less gradually decrease around the peaks. These peaks are not available during prediction on real data because we are trying to predict for locations that have not yet been experimentally verified. Therefore, any computational model must be able to predict for the peak as well as the surrounding non-peak regions. Further, the evaluation method must synthesize some criterion to determine what is the ground truth (is it an enhancer or not) for any genic region away from the peak location.

Consequently, RFECS introduced the notion of *validation, misclassification*, and *unknown* rates, to solve this problem. If a prediction is made that a location is an enhancer, RFECS says *the prediction is validated*, given that the location is sufficiently close to either a known peak marker or an open chromatin site (DHS) (2.5 kb to be precise) and sufficiently far from a TSS (1 kb to be precise). The second outcome is that *a prediction is misclassified* if the predicted location of an enhancer is too close to a TSS (2.5 kb to be precise). All other cases are considered as *prediction correctness is unknown*, i.e., there is no True Positive Marker or TSS within 2.5 kb of the predicted location of the enhancer.

We adopt the RFECS metrics, but make one improvement on it. The RFECS method singled out TSSs as misclassifications, while omitting known insulators, promoters, and other functional non-enhancer sites, and then lumping them together as ‘Unknown’. TSSs alone only make up a tiny portion of non-enhancers, which are not truly representative of the real overall misclassifications that a prediction algorithm makes. Furthermore, if enhancers are a subset of DHS, it is safe to assert that the unknown sites are, at the very least, *not enhancers* of any kind, and should be considered invalid as well. They should not be called “unknown”, from an enhancer prediction viewpoint since we “know” they are not enhancers. Rather, they should be labeled as “misclassification”.

Based on these observations, the RFECS validation method was refined to classify predicted enhancers as either “validated” or “invalidated”, using the following criteria. True Positive markers (TPM) refer to distal DHS sites, p300, CBP, and TFBS that are greater than 1 kb away from TSS.

- If a predicted enhancer lies within 2.5 kb of a TPM, then EP-DNN’s prediction is “validated”. In this case, we know that this site is either a known or an unknown enhancer, and can be safely assumed to be an enhancer since it overlaps with a DHS site.
- Otherwise, EP-DNN’s prediction is “invalidated”. This means that it is either a TSS or an Unknown, but we know for a fact it is *not* an enhancer.

### Runtime Measurements

The runtime of DNN, DEEP-EN, and RFECS for training and prediction were measured for 10 k, 20 k, 30 k, and 40 k samples each. Since actual run times are highly dependent on several factors, such as the level of parallelization, hardware, platform, or implementation language, each method’s runtime was measured as the CPU clock time, under the same environment implemented in MATLAB2014rb, with no parallelization. We wanted a fair comparison of all methods at its most basic algorithmic form, i.e., without giving an algorithm advantage due to a specific hardware acceleration. For example, since there are highly efficient computation platforms for training DNNs on GPUs (like Theano or TensorFlow), EP-DNN could have benefited from that, but that would have been a fair comparison with the other algorithms. Further, we acknowledge that some algorithms are more easily parallelizable than others and our method of using serial execution alone does not bring that aspect out. However, we followed this approach to take out the variability of different parallelization methods, which would have made it difficult to compare the runtime results of the different protocols.

## Results

### Validation Rate and Invalidity Rate Plots

**Figure 5:**
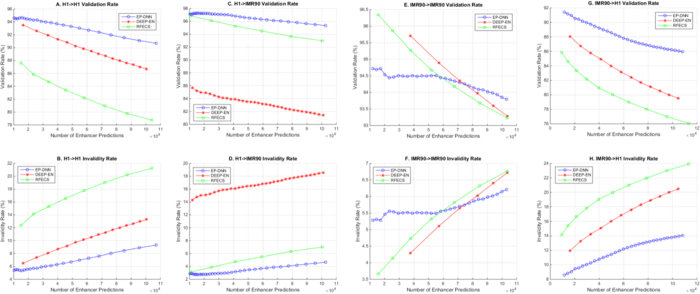
Plots showing the validation and invalidity rates for our algorithm (EP-DNN) and two recent algorithms that define the state-of-the-art, DEEP-EN and RFECS. These plots show the performance of these three algorithms for same-cell prediction (separately for H1 and IMR90 cell lines) and for cross-cell prediction (across these same two cell lines). (**A** and **B**) show the validation rate and invalidity rate of each method for enhancer prediction on an H1 cell-type using the same cell-type as a training set. (**C** and **D**) show the rates for cross-prediction on IMR90 using the same trained methods. (**E** and **F**) show the rates for same-cell prediction on IMR90. Finally, G and H show the cross-cell prediction rates for H1 with methods trained on IMR90 data.

Figure 5 shows the variation of validation and invalidity rates for the three protocols, when trained on the same datasets — our protocol EP-DNN and the two recent protocols, DEEP-EN and RFECS—for the two cell types, H1 and IMR90, for same-cell prediction as well as for cross-cell prediction. By varying the appropriate threshold parameter for each protocol, we are able to get a varying number of enhancer predictions. Table 3 summarizes the result, for a fixed number of enhancer predictions, at approximately 100,000 enhancer predictions, which appears to be a reasonable number of enhancers based on the ChIP-seq validated data points and falls squarely near the middle of the range prior work has mentioned (including DEEP-EN and RFECS). The first and most important observation is that EP-DNN performs better for validation and invalidity rates for both cell types, for same-cell and cross-cell predictions, across the entire range of number of enhancers being predicted (except for IMR90-IMR90 validation, where EP-DNN performs better for high number of enhancer predictions, which we explain later). Also note that the slope of the curve for EP-DNN is lower than for DEEP-EN and RFECS, implying that even when the protocol makes a large number of enhancer predictions, EP-DNN is more accurate. The only exception to the better performance of EP-DNN happens for IMR90 same-cell prediction, for high threshold values (*i.e.*, low number of predictions) where DEEP-EN and RFECS outperform EP-DNN. This likely happens because DEEP-EN and RFECS do a certain amount of overfitting to training data (DEEP-EN more so than RFECS) and such overfitting shows a (slightly) better prediction at high threshold values. This use case with high threshold values is arguably useful to experimentalists who are particular about high confidence predictions of enhancers for IMR90.

In addition, from the comparative validation rates for the different models (RFECS, DEEP-EN, EP-DNN), we gather the following cell-type and model-specific insights:

1. Same-cell prediction is easier than cross-cell prediction, as would be intuitive, with one exception. This is for the case that training on the H1 cell type and predicting on the IMR90, which turns out to be more accurate than IMR90 to IMR90 prediction when we use our EP-DNN model. This is potentially because H1 may be a harder cell type for training. Previous studies indicate that embryonic stem cells exhibit a richer set of variations within their histone modification signatures, stemming from the fact that they are enriched in transposable elements^42^,^43^, which are known to be enriched in active histone modifications, as per the exaptation hypothesis^44^. Further, it is possible that these signatures for the different classes of embryonic enhancers are more similar to each other than in non-embryonic stem cells^45^, possibly due to not being fully developed yet and having the potential to be developed into a wide variety of differentiated cell types^46^. Thus, once H1 is used as a training set via a powerful model that can capture the subtleties of the dataset (e.g., larger numbers of histone modification combinatorial codes representative of enhancers), it is able to achieve higher validation rates for cross-cell predictions. Thus, it indicates that our EP-DNN model is more powerful as a model to learn a classifier using a dataset where the positive and negative examples may be more “inter-mixed”, and thus, harder to classify. This underlines a fundamental motivation for our use of DNN—the increased power of the model, at the expense of a greater effort in tuning the algorithm. Further, given that the H1 cell type is an embryonic cell type that is formative in character, it stands to reason that the differences between the signatures of enhancers and non-enhancers may be harder to resolve in it. We can contrast this to the adult cell type (lung fibroblasts) used in our study, IMR90, where these differences while easier to resolve by a classifier, does not help in predicting enhancers in the embryonic cell type. This shows up in the comparatively lower validation rate for the IMR90 → H1 experiment. The conclusion from the above scenario can be summed up as follows: first, EP-DNN is a better learning model; second, the H1 cell type (and possibly by extrapolation any other embryonic cell type) presents a harder learning task; third, once this harder learning task is tamed, the insights gained by the model result in better accuracy for cross-cell prediction. If accurate cross-cell prediction were indeed possible, this would greatly reduce the need for conducting potentially lengthy and expensive experimentation on hitherto unseen cell types.
2. For RFECS, we see that the prediction is approximately equally accurate for same-cell and cross-cell predictions, as shown in Fig. 5 and Table 3. The absolute values are higher for IMR90 predictions than for H1, whether for same-cell or cross-cell prediction, attributed to the reasoning discussed above. This pertains to the smaller distance between positive and negative samples in H1. For DEEP-EN, there appears to be significant overfitting in that we see that the cross-cell prediction drops significantly for both cell types. This drop is greater than 10% toward the higher end of the number of enhancer predictions. However, in keeping with our speculation that H1 is a harder-albeit-better training set, we observe that cross-cell prediction numbers for IMR90 are higher than for H1, when trained on the other cell type, respectively.

### Validation Rate and Invalidity Rate Summary Table

We benchmark the validation and invalidity rates for the three techniques, ours namely EP-DNN, DEEP-EN, and RFECS, in Table 3. We keep the number of predictions by each technique to be close (approximately 100,000), for purposes of comparison. We find that DNN performs better in terms of both validation and invalidity rates. The advantage is more pronounced for prediction for the H1 cell type, where it is observed that enhancer prediction is a more difficult task than for IMR90. The improvement with DNN can be attributed to the use of the powerful DNN modeling technique, including multiple hidden layers and a large number of neurons at each layer, extensive feature selection, and optimization of the architecture and the parameters of the DNN. For cross cell prediction, it turns out that predicting IMR90 enhancers when the model is trained on H1 is an easier task than the opposite cross-cell prediction task, i.e., H1 → IMR90 is better than IMR90 → H1. The reasons for this have been discussed in the previous section.

### Validation Rate and Invalidity Rate Detailed Investigation

We investigate in greater detail the factors that contribute to the validation and invalidity rates and present the results in Tables 4 and 5. We find that the DHS that are distal from the TSS *and* the ones that are not p300, CBP, or TFBS (called *DHS-e*, “*e*” for enhancers), are the most numerous enhancers and provides the single largest contribution toward the validation rate. For IMR90, TFBS and CBP regions are not present in its dataset. The p300s and CBPs are more numerous in the data than the proportion in which they appear in our predictions. This can be explained by two factors. First, EP-DNN creates a model that generalizes well and does not overfit to the training data (which is all p300 for positive training examples) and consequently has a lower performance in predicting p300 sites. Second, the enrichment curves for p300s and CBPs are narrower, and thus, the signature may be weak toward the edge of the 2.5 kbp boundary from the enhancer peak location. For the invalidity rate, the single biggest contribution comes from incorrectly predicting the non-DHS regions as enhancers. Predicting some of the TSS as enhancers also contributes to the invalidity rate. The greatest contribution to the validation rate comes, as before, from the DHS regions that are *not* p300 binding sites but are enhancers. The greatest contribution to the invalidity rate, again, comes from predicting the non-DHS regions as enhancers. Note that we find that DNN is more prone to error in classifying some TSS sites as enhancers, more so than DEEP-EN and RFECS. However, the difference in TSS mis-prediction is not too significant between DNN and the others. The latter observation is borne out by the fact that the final invalidity rate for DNN is lower.

### Training and Prediction Time

The training time and, more importantly, the prediction time are two important qualities that determine the usability of predictive methods when dealing with large data sizes. We measured these times under a variety of experimental conditions—different numbers of samples and different numbers of histone modifications, and then tabulated the results in Table 6.

**Figure 6:**
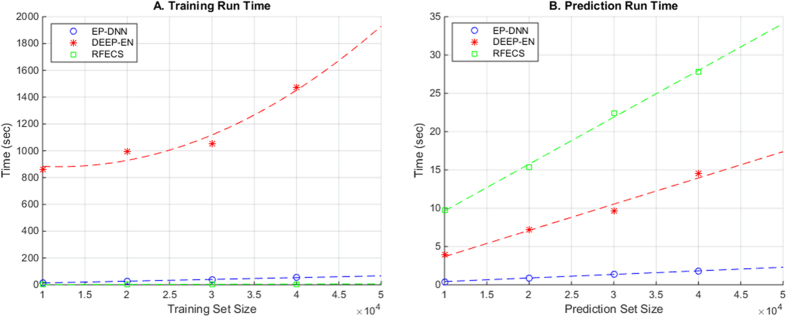
Training and prediction run time for the three protocols, EP-DNN (our protocol), RFECS, and DEEP-EN, for different sizes of the sample set. The curve fitting is done through a polynomial curve fit. (**A**) shows the training time where DEEP-EN takes a substantially longer time that EP-DNN or RFECS and also exhibits quadratic growth as the training set size increases, whereas the runtime of EP-DNN and RFECS are linear and the difference between the two are almost negligible. (**B**) shows the prediction runtime. EP-DNN has the fastest prediction time among the three methods.

EP-DNN and RFECS both show reasonably fast training times. However, we can see DEEP-EN is almost unusable without using specialized hardware and parallelization techniques to speed up training time (which we have not done here) (Fig. 6).

EP-DNN has the fastest prediction time of all the methods, while RFECS has the fastest training time. This is likely due to the use by RFECS of vectorized operations during training and creating the decision trees. Although this property allowed fast training for RFECS, it no longer applies during prediction since each decision tree node and each decision tree within the model has to be traversed one by one for each sample. This results in RFECS having the slowest run time when it comes to the prediction. Reflection on the results indicates the benefit in EP-DNN (from a runtime aspect) of not using an ensemble model, which keeps its computational cost bounded. We trade off the multiple layers of DNNs to handle the complexity in the patterns of the data. It turns out that computationally, this is a very worthwhile tradeoff, even with 5 layers (3 hidden layers, one input layer, and one output layer) in our DNN.

The fit in Fig. 6 also reveals that the training time for DEEP-EN increases exponentially with the training set size and thus, may be unusable at large training set sizes. For the prediction time, all three protocols show a linear trend, but the EP-DNN line has the lowest intercept as well as the lowest slope. Intercept indicates the fixed cost of the algorithm while the slope indicates the cost per sample. At the upper end of the range of prediction set size (40,000 samples), RFECS takes 30 seconds, DEEP-EN takes 15 seconds, while EP-DNN takes less than 2 seconds. The lowest slope for EP-DNN also means it will be usable at larger number of predictions.

## Discussion

In this section, we discuss the other beneficial aspects of using a DNN, which are not captured by the earlier quantitative results.

### Relation of EP-DNN to RFECS

We see from our experimental results that with respect to validation and invalidity rates that EP-DNN is faster and has superior performance than RFECS^34^. In addition, there is the issue of interpretability of the results of RFECS. With RFECS, a random forest is generated from multiple (65 in their final selection) decision trees. A decision tree has as an important quality that it is easily interpretable, but unfortunately, the power of a random forest comes at the cost of a decrease in the interpretability of the resultant model. Further, the use of the Fischer discriminant analysis at each node of RFECS makes it less interpretable. The final output of RFECS is a voting of the features as they appear in the constituent decision trees. However, the voting has to take two factors into account – the presence or absence of a feature in a specific tree plus where in the tree it appears (an appearance higher in the decision tree indicates higher importance). The result of the voting is a scalar value that is finally used to order the features, but the scalar value is not easily interpretable.

### Relation of EP-DNN to DEEP-ENCODE (DEEP-EN)

Another recent work that classifies DNA regions as enhancers or non-enhancers is DEEP-EN^35^. DEEP-EN’s key contribution is that it runs its classification on two new datasets, namely FANTOM5 and VISTA, which are significantly different from the ENCODE dataset. It also suffers from a lack of interpretability of the resulting model. DEEP-EN uses an ensemble of 1,000 Support Vector Machines (SVMs) for an intermediate classification result. It then uses an Artificial Neural Network (ANN). The inputs to this ANN are confidence scores (confidence scores are defined as the proportion of positive votes versus all votes for models from each cell line) obtained in the first layer of DEEP-EN from the four cell type-/tissue-specific ensemble models. The resultant model is therefore completely unintuitive to a human user and the paper also makes no claim about interpretability.

## Conclusion

In this paper, we have described the design and development of a deep learning based model, which we call EP-DNN, for predicting enhancers in epigenomic data using patterns of histone modifications. DNN with *softplus* units trained with *dropout* was used to predict enhancers in an embryonic cell type (H1) and a differentiated lung fibroblast cell type (IMR90). We demonstrated that DNNs work well in extracting features automatically from a set of eighty features in four histone modifications, and then, in using these to predict enhancers. We also showed that DNN predictions generalize well across different cell types (H1 → IMR90 and IMR90 → H1), especially when trained on the H1 cell type. Our experiments further suggest that embryonic stem cells have more tightly-packed data and can thus leverage higher model expressivity, specifically by affording the DNN classifier with the required data to come up with a more accurate decision boundary between the positive and negative examples. EP-DNN provides powerful feature extraction capabilities with relatively low computational cost.

Our work hints at the possibility of accurate prediction across cell types, once a model has been trained on a “complex” cell type, such as H1. Global prediction of enhancers will enable the rapid prediction of enhancers in new cell types, without the need for a separate training set for every new cell type. Once these putative enhancers in different cell types have been identified, it will be important to link them to the specific genic promoters that they regulate. This kind of interaction is complex with many-to-many associations, wherein one enhancer can regulate the expression of multiple genes, and multiple enhancers can affect the same gene, acting in synergy. Such predictions will lower the experimental cost of generating enhancer and gene interaction data through methods such as chromatin conformation capture-based protocols^47^.

Currently, the EP-DNN framework relies strongly on p300 binding sites and DHSs for positive training examples and is mostly centered around the selection of important histone modifications marking genomic enhancers. In the future, we will use data from other co-activator binding sites, potential sequence codes of enhancers^8^,^48^,^49^,^50^, DNA methylation^33^,^51^, and nucleosome destabilization data, in order to map enhancers more effectively. For negative training data, currently we have used random 100 bp bins that are distal to known p300 or TSS. With the emergence of more data for other types of chromatin elements, such as silencers, insulators, or extracellular matrix modifiers, these can be used, with higher certainty, for negative training. In addition, DNNs are capable of genome-wide mapping of these other types of chromatin elements, further annotating the genome-wide regulatory codes. What will also help is to integrate diverse types of datasets for the prediction of CRMs, as has been done in the EnhancerFinder model^52^, which has currently used the relatively small, albeit *in-vivo* validated, VISTA enhancer database^53^ for the prediction of developmental enhancers. In addition, linking different types of histone combinatorial codes that we have developed in this paper, with RNA-seq datasets that measure gene expression levels can potentially help classify enhancer activity levels, rather than the current binary classification.

## Additional Information

**How to cite this article**: Kim, S. G. *et al*. EP-DNN: A Deep Neural Network-Based Global Enhancer Prediction Algorithm. *Sci. Rep.* **6**, 38433; doi: 10.1038/srep38433 (2016).

**Publisher's note:** Springer Nature remains neutral with regard to jurisdictional claims in published maps and institutional affiliations.

## Supplementary Material

Supplementary Information

## Figures and Tables

**Table 1:** Comparison of different recent methods for enhancer prediction: EP-DNN, RFECS, DEEP-EN, ChromaGenSVM, CSI-ANN, and HMM.

	EP-DNN	RFECS	DEEP-EN	ChromaGenSVM	CSI-ANN	HMM
Feature Selection	4 histone modifications	3 histone modifications	11 histone modifications	~5 histone modifications	40+ histone modifications	10 histone modifications
Feature Extraction	DNN	FDA-based Random Forest	CSI-ANN method	Genetic Algorithm	FDA	Simulated Annealing
Hyperparameter Optimization	Cross validation	Manual ROC	Manual	Genetic Algorithm	Manual	Simulated Annealing
Classification	DNN	FDA-based Random Forest	SVM + ANN	SVM	Time Delayed ANN	Hidden Markov Model

**Table 2:** The number of peak calls of functional elements in the data set used for training and prediction, obtained through ChIP-seq and DNase-seq.

	H1 (100 bp)	IMR90 (100 bp)
**DHS**	150,729	149,787
**TSS**	9,299	8,000
**P300**	13,523	52,988
**CBP**	12,958	N/A
**TF**	71,173	N/A

**Table 3:** Validation rates for the three protocols—our protocol EP-DNN and the two recent algorithms that define the state-of-the-art, DEEP-EN and RFECS, where we keep the number of enhancer predictions approximately constant, at 100,000.

**H1→H1**	**Threshold**	**# of Predictions**	**Validation Rate (%)**	**Invalidity Rate (%)**
DNN	0.52	104,994	90.76	9.24
DEEP	83	105,030	86.43	13.57
RFECS	0.86	104,155	78.76	21.24
**H1→IMR90**				
DNN	0.64	100,632	95.40	4.60
DEEP	65	101,127	81.42	18.58
RFECS	0.92	100,344	93.00	7.00
**IMR90→IMR90**				
DNN	0.60	103,196	93.79	6.21
DEEP	94	103,751	93.28	6.72
RFECS	0.88	103,624	93.23	6.77
**IMR90→H1**				
DNN	0.56	97,178	86.26	13.74
DEEP	88	97,245	79.99	20.01
RFECS	0.80	95,174	77.00	23.00

This shows the validation rates for same-cell prediction and cross-cell prediction. Same cell prediction rates are higher, except for cross-cell prediction with training on H1, for EP-DNN.

**Table 4:** For prediction in the H1 cell type, a breakdown of the validation rate for the different components that are classified as positive: p300, CBP, Transcription Factors (NANOG, OCT4 and SOX2), and other DNase-I hypersensitive sites (DHS).

	Validation Rate (%)				Invalidity Rate (%)	
**H1→H1**	**p300**	**CBP**	**TFBS**	**DHS-e**^1^	**TSS**	**Non-DHS**
**EP-DNN**	0.28	0.25	15.69	74.54	3.56	5.68
**DEEP-EN**	0.38	0.26	26.94	58.85	1.11	12.46
**RFECS**	0.79	0.81	32.85	44.31	1.60	19.64
**IMR90→H1**	**p300**	**CBP**	**TFBS**	**DHS-e**	**TSS**	**Non-DHS**
**EP-DNN**	0.30	0.31	9.55	76.11	8.57	5.16
**DEEP-EN**	0.44	0.42	19.11	60.02	1.95	18.06
**RFECS**	0.84	0.81	30.90	44.45	1.55	21.45

A breakdown is also provided of the invalidity rate for the different components that are incorrectly classified as enhancers: transcription start sites (TSS) that overlap DNase-I, and random 100 bp bins that are distal to known p300 or TSS.

^1^We define *DHS-e* to be the DHS sites that are distal from TSS sites and are *not* p300, CBP, or TFBS.

**Table 5:** For prediction in the IMR90 cell type, a breakdown of the validation rate for the different components that are classified as positive: p300 and other DNase-I hypersensitive sites (DHS).

	Validation Rate (%)		Invalidity Rate (%)	
	p300	DHS-e	TSS	Non-DHS
**IMR90→IMR90**				
EP-DNN	19.41	74.38	2.45	3.76
DEEP-EN	16.93	76.35	0.69	6.02
RFECS	14.35	78.88	0.88	5.89
**H1→IMR90**				
EP-DNN	19.95	75.45	2.03	2.57
DEEP-EN	15.51	65.92	0.89	17.69
RFECS	13.97	79.02	0.69	6.31

A breakdown is also provided of the invalidity rate for the different components that are incorrectly classified as enhancers: transcription start sites (TSS) that overlap DNase-I, and random 100 bp bins that are distal to known p300 or TSS.

**Table 6:** The training and prediction time for EP-DNN, RFECS, and DEEP-EN measured in CPU cycles, without any parallelization applied.

DNN				
Sample size	Training time [4 mods] (s)		Prediction time [4 mods] (s)	
10 k	13.488305		0.397887	
20 k	27.002257		0.916081	
30 k	40.127233		1.367836	
40 k	53.499745		1.805095	
**RFECS**				
	Training time (s)		Prediction time (s)	
Sample size	24 mods	3 mods	24 mods	3 mods
10 k	2.486469	1.386131	10.491072	9.770147
20 k	4.651446	2.511087	16.876750	15.316730
30 k	6.729005	3.728762	25.762677	22.412472
40 k	9.241245	5.122801	29.065038	27.796367
**DEEP-EN**				
	Training time [11 mods] (s)		Prediction time [11 mods] (s)	
Sample size	Initialization & ANN	Training 1 SVM	100 SVMs	
10 k	10.854171	8.507385	3.932895	
20 k	8.439214	9.857070	7.159999	
30 k	12.011724	10.404023	9.657686	
40 k	16.891985	14.569916	14.515174	
