# Supplementary material for: EP-DNN: A Deep Neural Network-Based Global Enhancer Prediction Algorithm
Source: Sci Rep. 2016 Dec 8;6:38433. doi: 10.1038/srep38433 (PMC5144062; doi:10.1038/srep38433)
Supplement: Supplementary Information [file srep38433-s1.pdf]

EP-DNN: A Deep Neural Network-Based Global Enhancer Prediction Algorithm

Seong Gon Kim, Mrudul Harwani, Ananth Grama, Somali Chaterji

Department of Computer Science, Purdue University

Correspondence to schaterj@purdue.edu

SUPPLEMENTARY

We also carry out an investigation in which we change the threshold for the three different protocols—EP-DNN, DEEP-EN, and RFECS – and observe the effect on the validation and invalidity rates.

The detailed results are included here, separately for each of the two same-cell predictions (H1 and IMR90), ***Table S7 and S9***, and for each of the two cross-cell predictions (IMR90 → H1 and H1→ IMR90) in ***Tables S8 and S10***. The outputs from each algorithm can be interpreted as a confidence level. The higher the threshold, the more confident the algorithm is that that particular location is an enhancer. However, the algorithms have different scales for thresholds. For example, the level of confidence from an output of 0.5 from EP-DNN is different from an output of 0.5 from DEEP-EN (since DEEP-EN is a majority vote). The maximum validation rate in the table is the highest for EP-DNN in all cases. Even when finding a small number of enhancers (and keeping this number constant across the three protocols), EP-DNN has higher validation rate. This means it can find higher confidence enhancer site more accurately.

Training with IMR90 on EP-DNN gives a maximum output less than 0.8 and hence, threshold values 0.8 and above are not available (N/A). This means the EP-DNN is biased toward the zero value since it never fully reaches an output of 1 (it never even reaches an output of 0.8). This can be interpreted as lower confidence on the elements it is predicting as an enhancer. This only happens when using IMR90 as training data, not for H1 training data. So, we conclude that IMR90 does not contain information that fully captures the subtleties of enhancer signatures. Consequently, EP-DNN gives less accurate predictions when trained on IMR90. Therefore, IMR90 is not as good as a training set as H1. We hypothesized this earlier and provided some evidence. This result adds to the body of evidence.

Here we see the effect of varying the threshold to the validation rate and the invalidity rate, for each of the three protocols—EP-DNN, DEEP-EN, and RFECS. A threshold value of k for EP-DNN means that if the output of EP-DNN is greater than k, then that sample is considered an enhancer. A similar definition is used for DEEP-EN and RFECS. As the threshold is increased, the number of (positive) predictions naturally decreases, since the criteria for something to be called an enhancer is being made stricter. For EP-DNN, with increasing threshold, the validation rate improves till the very end, when there is a slight dip (threshold going from 0.8 to 0.9). The increase in validation rate is explained by the fact that a fewer number of samples are now being labeled as enhancers, but the ones that are being flagged as enhancers are more likely to be enhancers. Thus, in the formula for validation rate, the denominator (the number of samples predicted to be enhancers) decreases and so does the numerator (of the positive samples predicted, how many are actually predicted). But the decrease in the denominator is less sharp, leading to an overall increase in validation rate. However, beyond a certain threshold (somewhere between 0.8 and 0.9) there are far too many genuine enhancers that are being missed and consequently, the validation rate dips a little. For both DEEP-EN and RFECS, as the thresholds are increased, the validation rate keeps increasing. The dip toward the very end is not seen and this is likely because the jumps in threshold are too big. .

With increasing threshold, the invalidity rate monotonically decreases for all three protocols. This can be explained by the fact that the criteria for calling something an enhancer is becoming stricter with increasing threshold values. Thus, there is less likelihood that something is incorrectly being predicted to be an enhancer. Mathematically, for the invalidity rate, with increasing threshold, the number of samples that are being predicted as enhancers (the denominator) decreases, as does the number of those samples in the numerator that are actually TSS or unknowns (the numerator). But here the numerator decreases faster than the numerator, causing the overall invalidity rate to go down.

A reasonable setting for the thresholds will make a sizeable number of positive predictions, say 100K samples. Thus, really large values of the threshold are not very useful because too few of the enhancers re being found out.

Table S1. Same-cell prediction for the H1 cell type: Variation of validation rate and invalidity rate when the protocol-specific threshold is varied, individually for EP-DNN, DEEP-EN, and RFECS. For EP-DNN and RFECS, the range for the threshold is [0, 1] and for DEEP-EN, it is [0, 100].

**DNN H1→H1**			
**Threshold**	**# of Predictions**	**Validation Rate (%)**	**Invalidity Rate (%)**
0	30,946,020	10.9	89.1
0.1	3,319,245	43.16	56.84
0.2	2,624,875	48.51	51.49
0.3	1,740,701	57.62	42.38
0.4	483,864	79.53	20.47
0.5	136,338	89.54	10.46
0.6	42,861	93.56	6.44
0.7	14,813	94.65	5.35
0.8	5,320	94.51	5.49
0.9	1,860	93.71	6.29
**DEEP H1→H1**			
**Threshold**	**# of Predictions**	**Validation Rate (%)**	**Invalidity Rate (%)**
0	1,605,366	49.37	50.63
10	596,670	65.71	34.29
20	455,362	70.13	29.87
30	369,648	73.21	26.79
40	305,330	75.82	24.18
50	251,665	78.22	21.78
60	204,587	80.5	19.5
70	160,603	82.98	17.02
80	117,783	85.62	14.38
90	72,648	88.71	11.29
**RFECS H1→H1**			
**Threshold**	**# of predictions**	**Validation Rate (%)**	**Invalidity Rate (%)**
0	10,406,399	22.73	77.27
0.1	3,305,770	40.52	59.48
0.2	1,946,295	48.67	51.33
0.3	1,390,667	53.53	46.47
0.4	976,822	58.48	41.52
0.5	725,931	62.38	37.62
0.6	496,447	66.7	33.3

0.7	327,142	70.48	29.52
0.8	161,016	75.96	24.04
0.9	57,983	82.23	17.77

Table S2. Same-cell prediction for the IMR90 cell type: Variation of validation rate and invalidity rate when the protocol-specific threshold is varied, individually for EP-DNN, DEEP-EN, and RFECS. For EP-DNN and RFECS, the range for the threshold is [0, 1] and for DEEP-EN, it is [0, 100].

**DNN IMR90→IMR90**			
**Threshold**	**# of predictions**	**Validation Rate (%)**	**Invalidity Rate (%)**
0	30,939,249	17.18	82.82
0.1	2,860,770	65.89	34.11
0.2	2,567,110	68.29	31.71
0.3	2,184,051	71.42	28.58
0.4	619,534	86.56	13.44
0.5	246,489	91.47	8.53
0.6	103,196	93.79	6.21
0.7	1,618	95.74	4.26
0.8	0	NaN	NaN
0.9	0	NaN	NaN
**DEEP IMR90→IMR90**			
**Threshold**	**# of predictions**	**Validation Rate (%)**	**Invalidity Rate (%)**
0	1,549,404	72.42	27.58
10	763,248	84.05	15.95
20	603,683	85.96	14.04
30	504,074	87.07	12.93
40	427,812	87.95	12.05
50	364,072	88.72	11.28
60	307,113	89.46	10.54
70	253,738	90.2	9.8
80	200,045	91.06	8.94
90	136,579	92.39	7.61
**RFECS IMR90→IMR90**			
**Threshold**	**# of predictions**	**Validation Rate (%)**	**Invalidity Rate (%)**
0	6,429,462	52.24	47.76
0.1	2,842,624	67.97	32.03
0.2	1,925,611	72.86	27.14
0.3	1,465,397	76.01	23.99
0.4	1,079,653	79.31	20.69
0.5	822,667	81.99	18.01

0.6	570,131	85.16	14.84
0.7	381,161	88	12
0.8	191,744	91.31	8.69
0.9	67,532	94.18	5.82

Table S3. Cross-cell prediction when trained on the H1 cell type and predicting on the IMR90 cell type: Variation of validation rate and invalidity rate when the protocol-specific threshold is varied, individually for EP-DNN, DEEP-EN, and RFECS. For EP-DNN and RFECS, the range for the threshold is [0, 1] and for DEEP-EN, it is [0, 100].

**DNN H1→IMR90**			
**Threshold**	**# of predictions**	**Validation Rate (%)**	**Invalidity Rate (%)**
0	30,949,205	17.19	82.81
0.1	2,802,016	70.18	29.82
0.2	2,428,544	73.13	26.87
0.3	1,873,492	77.55	22.45
0.4	776,977	87.11	12.89
0.5	316,528	92.06	7.94
0.6	140,468	94.6	5.4
0.7	64,987	96.21	3.79
0.8	30,752	97.08	2.92
0.9	14,532	97.25	2.75
**DEEP H1→IMR90**			
**Threshold**	**# of predictions**	**Validation Rate (%)**	**Invalidity Rate (%)**
0	1,953,672	56.13	43.87
10	443,863	70.4	29.6
20	315,026	74.05	25.95
30	241,867	76.37	23.63
40	190,581	78.14	21.86
50	150,335	79.56	20.44
60	116,489	80.83	19.17
70	86,676	82.03	17.97
80	59,613	83.2	16.8
90	32,633	84.12	15.88
**RFECS H1→IMR90**			
**Threshold**	**# of predictions**	**Validation Rate (%)**	**Invalidity Rate (%)**
0	6,911,985	49.34	50.66
0.1	3,261,852	65.31	34.69
0.2	2,296,602	70.74	29.26
0.3	1,788,360	73.77	26.23
0.4	1,324,765	76.96	23.04
0.5	1,003,928	79.68	20.32

0.6	711,326	82.6	17.4
0.7	504,981	84.96	15.04
0.8	284,220	88.23	11.77
0.9	123,277	92.25	7.75

Table S4. Cross-cell prediction when trained on the H1 cell type and predicting on the IMR90 cell type: Variation of validation rate and invalidity rate when the protocol-specific threshold is varied, individually for EP-DNN, DEEP-EN, and RFECS. For EP-DNN and RFECS, the range for the threshold is [0, 1] and for DEEP-EN, it is [0, 100].

**DNN IMR90→H1**			
**Threshold**	**# of predictions**	**Validation Rate (%)**	**Invalidity Rate (%)**
0	30,942,335	10.9	89.1
0.1	3,839,298	36.48	63.52
0.2	3,235,142	39.95	60.05
0.3	2,509,038	45.17	54.83
0.4	421,642	77.49	22.51
0.5	157,745	84.88	15.12
0.6	69,523	87.29	12.71
0.7	74	85.14	14.86
0.8	0	N/A	N/A
0.9	0	N/A	N/A
**DEEP IMR90→H1**			
**Threshold**	**# of predictions**	**Validation Rate (%)**	**Invalidity Rate (%)**
0	2,071,714	40.42	59.58
10	946,081	55.37	44.63
20	715,431	59.93	40.07
30	573,721	63.14	36.86
40	466,472	65.85	34.15
50	377,732	68.4	31.6
60	298,497	70.92	29.08
70	226,496	73.62	26.38
80	155,641	76.72	23.28
90	81,902	81.11	18.89
**RFECS IMR90→H1**			
**Threshold**	**# of predictions**	**Validation Rate (%)**	**Invalidity Rate (%)**
0	12,357,935	20.44	79.56
0.1	4,134,960	35.66	64.34
0.2	2,228,668	45.48	54.52
0.3	1,494,843	51.51	48.49
0.4	976,482	57.15	42.85
0.5	677,755	61.45	38.55

0.6	419,315	66.35	33.65
0.7	245,961	70.95	29.05
0.8	95,174	77	23
0.9	21,393	83.37	16.63
